# Prognostic Impact of Chronic Kidney Disease After Percutaneous Coronary Intervention with Drug-Coated Balloons

**DOI:** 10.3390/jcm14072317

**Published:** 2025-03-28

**Authors:** Tetsuya Takahashi, Tetsu Watanabe, Mashu Toyoshima, Wataru Katawaki, Taku Toshima, Yu Kumagai, Tamon Yamanaka, Masafumi Watanabe

**Affiliations:** 1 The Department of Cardiology, Japanese Red Cross Ishinomaki Hospital, Ishinomaki 986-0861, Japan; 2 The Department of Cardiology, Pulmonology, and Nephrology, Yamagata University School of Medicine, Yamagata 990-9585, Japan

**Keywords:** drug-coated balloon, chronic kidney disease, prognosis

## Abstract

**Background**: A drug-coated balloon (DCB) is an emerging treatment technology for percutaneous coronary intervention (PCI). However, the prognostic factors of PCI with a DCB remain fully determined. Chronic kidney disease (CKD) is an independent predictor of adverse outcomes in patients with coronary artery disease (CAD) who underwent PCI. The aim of this present study was to clarify the impact of CKD on prognosis in CAD patients who underwent PCI with a DCB. **Methods**: We enrolled 252 consecutive patients with CAD who underwent PCI with a DCB from 2015 to 2023. The endpoints of this study were composite events including all-cause death, myocardial infarction, target vessel revascularization, stroke, and major bleeding. **Results**: The prevalence rate of CKD was 48%. Patients with CKD were older and had higher prevalence of hypertension and diabetes mellitus than those without. Kaplan–Meier analysis revealed a significantly higher composite event rate in patients with CKD (log-rank test, *p* = 0.003). In the multivariate Cox proportional hazards analysis, CKD was independently associated with composite events after adjusting for confounding factors (adjusted hazard ratio 1.985, 95% confidence intervals 1.157–3.406, *p* = 0.013), mainly driven by all-cause deaths. **Conclusions**: CKD was associated with unfavorable outcomes in CAD patients who underwent PCI with a DCB.

## 1. Introduction

Drug-coated balloons (DCBs) have been established as a novel treatment option of percutaneous coronary intervention (PCI) [1,2,3]. The previous study has demonstrated that DCB treatment has been non-inferior compared to conventional drug-uncoated balloon angioplasty for the treatment of in-stent restenosis [1]. Use of a DCB has also shown comparable clinical outcomes compared to a drug-eluting stent (DES) in coronary artery disease (CAD) patients with small vessels [2,3]. Furthermore, recent studies have demonstrated the favorable clinical effect of DCBs in CAD patients with multi-vessels, de novo large vessels, and long diffuse vessels [4,5,6]. However, the prognostic impact of a DCB in patients with CAD has not been adequately addressed, although there are several favorable clinical data in the setting of comparison between a DCB and a DES.

Chronic kidney disease (CKD) is reportedly observed in approximately 10% of the general population [7]. The prevalence rate of CKD in patients with CAD has been increasing with aging population [8,9]. The previous study demonstrated that CAD severity was associated with the CKD [10]. The presence of CKD has been associated with unfavorable prognosis in patients with CAD [11]. Therefore, CKD has been recognized as one of the important predictor for cardiovascular prognosis in the era of DESs [12]. However, the association between CKD and DCBs remains unclear.

The aim of the present study was to clarify the prognostic impact of CKD on prognosis in patients with CAD who had PCI performed using a DCB.

## 2. Methods

### 2.1. Study Population

This is a prospective, single-center, observational study. Two hundred and seventy-two consecutive CAD patients who had PCI performed with a DCB between January 2015 and December 2023 were enrolled in the present study. Twenty patients were excluded due to missing clinical data after PCI. We analyzed the residual 252 patients in this study.

Acute coronary syndrome (ACS) and chronic coronary syndrome (CCS) were diagnosed based on the current guidelines, respectively [13,14].

Coronary artery stenosis was evaluated in angiography according to the current guideline of the American College of Cardiology/American Heart Association/Society for Cardiovascular Angiography and Interventions (ACC/AHA/SCAI) [15]. Visually evaluated diameter stenosis of ≥70% for non-left main disease and ≥50% for left main disease are defined as significant coronary stenosis.

PCI was undertaken in accordance with current standard techniques and guidelines [16,17,18]. DCB treatment was performed according to a contemporary statement [19,20]. For culprit lesion modification, balloon angioplasty was performed in advance of DCB treatment. After balloon angioplasty, DCB treatment was performed if there were no flow-limiting dissections and >30% visual residual stenosis. In other cases, a DES could be chosen at the PCI operator’s discretion.

Clinical, demographic, and interventional data on age, sex, smoking, atrial fibrillation (AF), family history of CAD, previous myocardial infarction (MI), previous PCI, previous coronary artery bypass graft (CABG), previous stroke, chronic heart failure (CHF), clinical presentation of peripheral artery disease (PAD), abdominal aortic aneurysm (AAA), chronic obstructive pulmonary disease (COPD), a malignant tumor, ACS, and CCS were collected from electronic medical records and examinations. Medications of each patient were also collected from electronic medical records and examinations at discharge. Body mass index (BMI) was derived during hospitalization. Hypertension, diabetes mellitus, and dyslipidemia were diagnosed on the basis of medical records or a history of related medical therapy. Transthoracic echocardiography was performed by physicians. Venous blood samples were obtained early in the morning within 24 h of admission.

The study was conducted according to the guidelines of the Declaration of Helsinki and approved by the Institutional Review Board of the Japanese Red Cross Ishinomaki Hospital (approval number: 22–23, and date of approval: 6 March 2023). The protocol of the present study was designed following the Strengthening the Reporting of Observational Studies in Epidemiology (STROBE) guidelines.

### 2.2. Assessment of Renal Function

The eGFR was calculated based on the Modification of Diet in Renal Disease equation with the Japanese coefficient [21]. Serum creatinine levels were measured using routine laboratory methods. Chronic kidney disease (CKD) was defined as reduced eGFR (<60 mL/min/1.73 m^2^) according to Kidney Disease Outcomes Quality Initiative clinical guidelines [22].

### 2.3. Endpoints and Follow-Up

Patients were prospectively followed-up for a median duration of 492 days (interquartile range 248–1532 days). Clinical follow-up data were obtained from outpatient record reviews and telephone interviews. The primary endpoint of the present study was a composite event including all-cause death, myocardial infarction, target vessel revascularization, stroke, and major bleeding (Bleeding Academic Research Consortium [BARC] type 3–5) [23]. The secondary endpoints were each component of the primary endpoint. Target vessel revascularization is defined as any repeat PCI or surgery with CABG of the target lesion for ischemic symptoms and events.

### 2.4. Statistical Analysis

The Shapiro–Wilk test was used to assess the normality of continuous variables. Mean ± standard deviation (SD) was used for continuous data, and medians with interquartile ranges were used for skewed data. Continuous and categorical variables were compared using unpaired Student’s *t*-test and Chi-squared test, respectively. The Mann–Whitney U-test was used for skewed data. Univariate and multivariate analyses with Cox proportional hazards regression were used to determine significant predictors of clinical events. Age, sex, and significant predictors in the univariate analysis were entered into the multivariate analysis. The Kaplan–Meier method and the log-rank test were performed to assess cumulative overall and event-free survival rates. *p* < 0.05 was regarded as statistical significance. We performed all statistical analyses using R studio version 2023.12.1+402.

## 3. Results

### 3.1. Baseline Characteristics

The baseline clinical characteristics of the 252 CAD patients who had PCI performed using a DCB are shown in Table 1. The mean age was 68.8 ± 11.9 years, and there were 204 men (81%). Hypertension, dyslipidemia, diabetes mellitus, smoking, and AF were identified in 208 (83%), 142 (56%), 116 (46%), 172 (68%), and 39 (15%), respectively. Family history of CAD, previous MI, previous PCI, previous CABG, previous stroke, CHF, PAD, AAA, COPD, and a malignant tumor were identified in 27 (11%), 58 (23%), 96 (38%), 10 (4%), 32 (13%), 61 (24%), 20 (8%), 14 (6%), 18 (7%), and 36 (14%), respectively. ST elevation MI (STEMI), non-STEMI (NSTEMI), and unstable angina were observed in 30 (12%), 54 (21%), and 14 (6%) patients, respectively. There were 154 (61%) patients with CCS. Three vessel disease and left main disease were identified in 35 (14%) and 11 (4%) patients, respectively. The prevalence of chronic total occlusion (CTO) was 37 (15%). Sixty patients were treated in combination with a DES. The mean left ventricular ejection fraction was 54.1 ± 13.5%. The mean serum levels of sodium, potassium, and chloride were 140.1 ± 3.0 mmol/L, 4.3 ± 0.5 mmol/L, and 104.6 ± 3.1 mmol/L, respectively.

Patients were divided into two groups in accordance with the prevalence of CKD. Patients with CKD were significantly older and the group contained more women than in the group without CKD. The prevalence of hypertension, diabetes mellitus, previous PCI, previous CABG, previous stroke, and CHF were significantly higher in patients with CKD than in those without CKD. Patients with CKD had lower prevalence rates of smoking and family history of CAD. CCS, three vessel diseases, and CTO were more likely observed in patients with CKD than in those without CKD. LVEF was significantly lower in patients with CKD than in those without CKD. Serum potassium concentration was significantly higher in patients with CKD than in those without CKD. Patients with CKD took more β blockers than those without CKD. There were no significant differences in the other clinical factors between the two groups (Table 1).

### 3.2. Association Between CKD and Clinical Outcomes

During the follow-up period, there were 70 composite clinical events, including 19 all-cause deaths, 3 cardiovascular deaths, 12 MI, 34 target vessel revascularization, 4 stroke, and 1 instance of bleeding.

**Figure 1:**
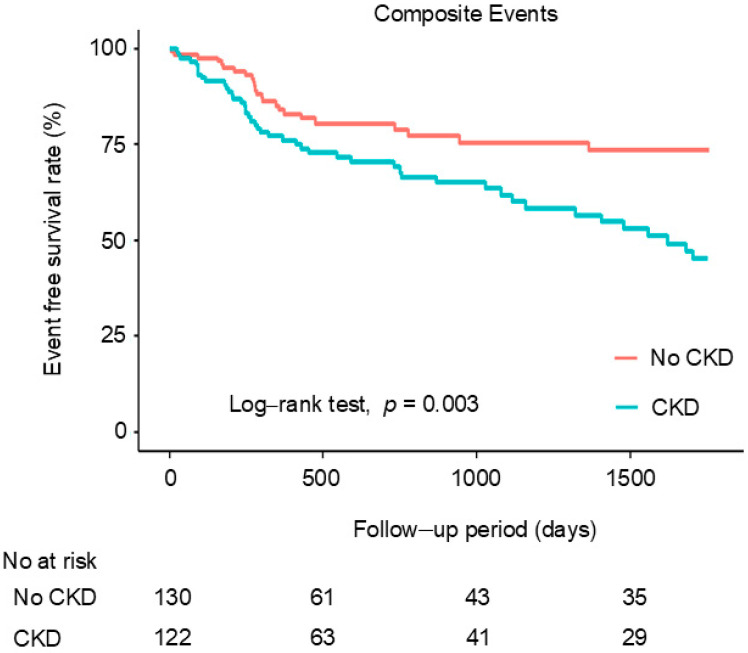
Cumulative incidence of composite events according to CKD status. CKD, chronic kidney disease.

The Kaplan–Meier analysis demonstrated that composite clinical events were higher in patients with CKD who underwent PCI with a DCB than in those without CKD (log-rank test, *p* = 0.003) (Figure 1). In the subgroup analysis, CKD was significantly associated with poor prognosis in patients with CCS (log-rank test, *p* = 0.003) but not in those with ACS (log-rank test, *p* = 0.300) (Supplemental Figure S1A,B).

**Figure 2:**
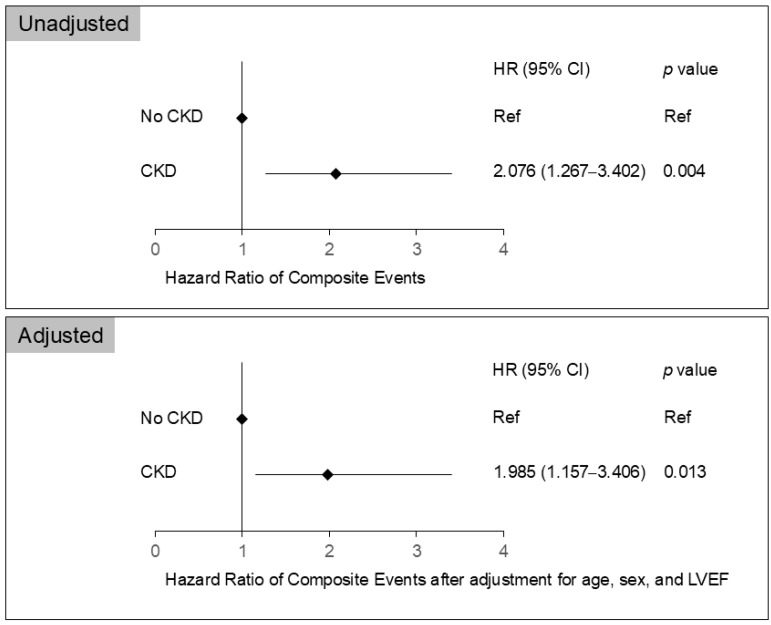
Univariate and multivariate Cox proportional hazard regression analyses for composite events according to CKD status. CKD, chronic kidney disease.

In the univariate Cox proportional hazard analysis, CKD and LVEF were significantly associated with the composite events. Multivariate Cox proportional hazard analysis showed that CKD was independently associated with the composite events after adjusting for age, sex, and LVEF (hazard ratios, 1.985; 95% confidence intervals, 1.157–3.406; *p* = 0.013) (Figure 2).

**Figure 3:**
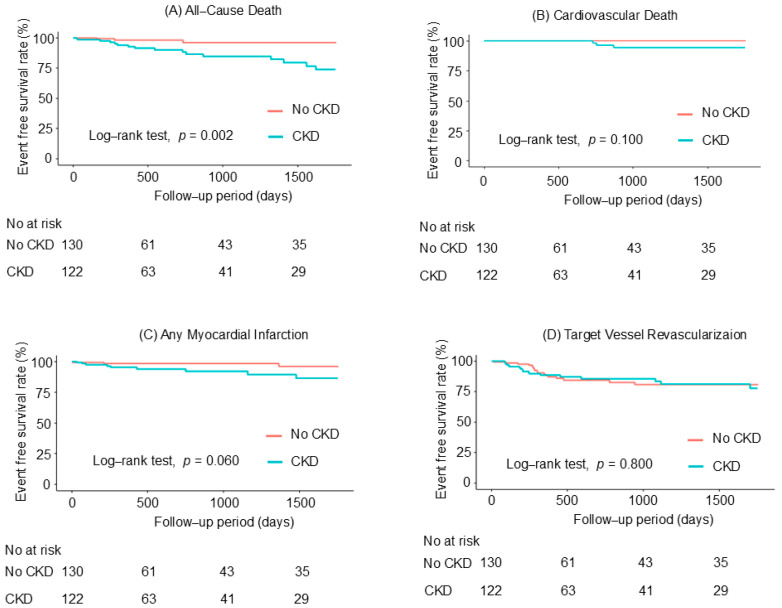

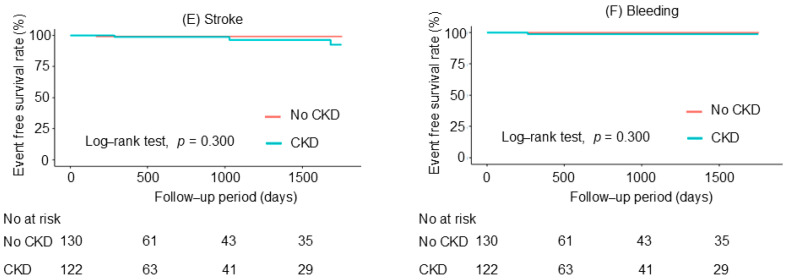
Cumulative incidence of (**A**) all-cause death, (**B**) cardiovascular death, (**C**) any myocardial infarction, (**D**) target vessel revascularization, (**E**) stroke, and (**F**) bleeding according to CKD status. CKD, chronic kidney disease.

The higher composite event risk was mainly driven by an increased risk of all-cause death (Figure 3 and Table 2).

Further, the patients were divided into five groups according to the CKD stages classified by eGFR: G1 (≥90 mL/min/1.73 m^2^, *n* = 11), G2 (60–89 mL/min/1.73 m^2^, *n* = 119), G3 (30–59 mL/min/1.73 m^2^, *n* = 88), G4 (15–29 mL/min/1.73 m^2^, *n* = 4), G5 (<15 mL/min/1.73 m^2^, *n* = 30) [22]. Kaplan–Meier analysis revealed that composite events were increased with advancing CKD stages (log-rank test, *p* < 0.0001) (Supplemental Figure S1).

## 4. Discussion

In the present study, (1) Kaplan–Meier analysis demonstrated a significantly higher event rate in patients with CKD than in those without; and (2) multivariate Cox hazard analysis revealed that CKD was significantly associated with poor prognosis in CAD patients who had PCI performed with a DCB, driven by higher all-cause mortality.

Recently, outcomes of PCI have been dramatically improved since various technologies of interventional cardiology have been developed. Especially, the evolution of DESs and procedural techniques and the use of intravascular imaging have made a great contribution to improvement of outcomes of PCI [24]. Furthermore, optimal short duration of dual antiplatelet therapy (DAPT) after PCI has succeeded in favorable prognosis [25]. However, there are still residual risks of PCI such as stent-related adverse events in the late phase after PCI even in the era of contemporary DESs. A recent study demonstrated that very-late stent-related events developed 1 to 5 years after PCI regardless of the type of stents, and the rate of events was about 2% per year with no plaque evident [26]. Contemporary stent technologies have not succeeded in the conquest of this ongoing very-late stent-related event risks [26]. Stent under-expansion, malposition, uncovered stent strut, hypersensitivity reactions, stent fracture, and neoatherosclerosis have been reportedly associated with very-late stent-related events [27]. Therefore, a treatment strategy that leaves nothing in the coronary artery is needed.

A DCB is a novel technology for PCI and a well-established treatment option for contemporary PCI. A DCB directly releases antiproliferative drugs into the vessel wall of the coronary artery, which leads to a reduction in the untoward effects associated with polymer-based stent technologies [28]. In the clinical setting of coronary in-stent restenosis, treatment with a DCB significantly reduced the adverse events and recurrent in-stent restenosis compared to a drug-uncoated balloon [1]. For the treatment of small native coronary arteries, DCBs also showed non-inferior event rates compared to DESs with respect to major adverse cardiovascular events (MACE) for up to 3 years [2,3]. Recently, DCB treatment in combination with a DES in CAD patients with multivessel diseases significantly reduced MACE at 2 years [4]. Furthermore, recent studies have demonstrated that a DCB has a potential to improve the cardiovascular outcomes compared to a DES even in de novo large coronary arteries [5]. Thus, DCBs have various favorable impacts on prognosis in CAD patients who had PCI performed in the era of contemporary DESs. However, the prognostic factors of DCBs in CAD patients who underwent PCI have not been fully described. Further, the favorable clinical evidences about DCBs are almost in the setting of the comparison between DCBs and DESs, as described above [1,2,3,4,5].

In the present study, we demonstrated that CKD was associated with poor prognosis in CAD patients who had PCI performed using a DCB. The prevalence rate of CKD in patients with CAD who had PCI performed has been increasing due to the aging population [8]. CAD patients with CKD showed worse prognosis than those with preserved renal function [11]. A recent study demonstrated that angiographic complete revascularization using a DES led to favorable prognosis in CAD patients with CKD [29]. However, there are few data about the prognostic impact of CKD on PCI with a DCB. The subgroup analysis of BASKET-SMALL 2 demonstrated that patients with renal disease who underwent PCI using a DCB tended to have higher MACEs than those without, although the prognoses were not significantly different between DCBs and DESs [2]. Similarly to these results of subgroup analysis, our study further provides the association between the presence of CKD and unfavorable prognosis in CAD patients who underwent PCI with a DCB.

The pathophysiological role of CKD on the poor clinical outcomes of CAD patients who underwent PCI using a DCB has not been fully addressed. The presence of CKD was reported to be associated with the risk of increased coronary lesion complexity in patients with CAD [10]. Furthermore, inflammation has been reported to initiate CAD in patients with CKD in addition to traditional coronary artery risk factors [10,30,31]. In the present study, the proportion of severe CAD status such as three vessel disease and CTO was significantly higher in patients with CKD than in those without. In CAD patients who underwent PCI with a DCB, the presence of CKD might affect the progression of CAD, which leads to poor clinical outcomes.

In the present study, the poor prognosis in CKD patients was mainly driven by an increased risk of all-cause death. In a previous study of PCI with DESs, the all-cause mortality in CKD patients was about 10% [29], which was consistent with the results of this study. The incident any MI was also higher in patients with CAD, although it did not reach statistical significance. This result suggested that the presence of CKD may accelerate atherosclerotic lesion progression and lead to unfavorable prognosis after PCI with a DCB.

## 5. Study Limitations

There are several limitations in the present study. First, this was a single-center study with a relatively small sample size. The statistical power for predicting clinical hard endpoints was weak since the number of events was small and the formal sample size was missing. Further, there was a possibility of misclassification bias, unmeasured confounding, and type 1 + 2 error. A large study population is needed to further clarify prognostic factors in patients with CAD who underwent PCI with a DCB, although the sample sizes of recent studies about DCBs were relatively small [4,5]. Second, there were no detailed data of the procedure of PCI, such as length and diameter of DCBs, length of culprit lesion, and contrast volume, because the present study did not focus on the PCI procedure. Since the presence of CKD has reportedly been associated with the progression of atherosclerosis [10], the PCI procedure might be different between two groups with and without CKD, which might affect clinical outcomes in the present study. Third, the data of complete revascularization was missing in the present study. The previous study demonstrated that complete revascularization was associated with favorable prognosis in CAD patients with CKD who had PCI performed with a DES [29]. There is a possibility that the presence of complete revascularization might affect the results in the present study. Fourth, we did not analyze the data of intracoronary imaging, although the usage rate of intracoronary imaging devices was 98% in the present study. The detailed data of intracoronary imaging devices might provide important information about the role of CKD on unfavorable clinical outcomes in CAD patients who had PCI performed using a DCB. Further studies are required to reveal the extent of these limitations.

## 6. Conclusions

CKD was associated with unfavorable clinical outcomes in patients with CAD who underwent PCI with a DCB. The presence of CKD provides useful information for the risk stratification of CAD patients who underwent PCI with a DCB.

## Figures and Tables

**Table 1:** Baseline clinical characteristics.

	All Patients (*n* = 252)	No CKD (*n* = 130)	CKD (*n* = 122)	*p* Value
Age, years	68.8 ± 11.9	66.8 ± 12.3	70.8 ± 11.2	0.007
Males, *n* (%)	204 (81)	115 (88)	89 (73)	0.003
Body mass index, kg/m^2^	24.9 ± 4.0	25.4 ± 3.7	24.4 ± 4.2	0.059
Hypertension, *n* (%)	208 (83)	97 (75)	111 (91)	0.001
Dyslipidemia, *n* (%)	142 (56)	73 (56)	69 (57)	1.000
Diabetes mellitus, *n* (%)	116 (46)	47 (36)	69 (57)	0.002
Smoking, *n* (%)	172 (68)	97 (75)	75 (62)	0.035
AF, *n* (%)	39 (15)	19 (15)	20 (16)	0.829
Family history of CAD, *n* (%)	27 (11)	20 (15)	7 (6)	0.023
Previous MI, *n* (%)	58 (23)	23 (18)	35 (29)	0.055
Previous PCI, *n* (%)	96 (38)	41 (32)	55 (45)	0.037
Previous CABG, *n* (%)	10 (4)	1 (1)	9 (7)	0.018
Previous Stroke, *n* (%)	32 (13)	10 (8)	22 (18)	0.023
Chronic heart failure, *n* (%)	61 (24)	12 (9)	49 (40)	< 0.001
PAD, *n* (%)	20 (8)	9 (7)	11 (9)	0.703
AAA, *n* (%)	14 (6)	5 (4)	9 (7)	0.343
COPD, *n* (%)	18 (7)	11 (9)	7 (6)	0.552
Malignant tumor, *n* (%)	36 (14)	19 (15)	17 (14)	1.000
Clinical presentation, *n* (%)				
STEMI	30 (12)	19 (15)	11 (9)	0.239
NSTEMI	54 (21)	31 (24)	23 (19)	0.417
Unstable angina	14 (6)	10 (8)	4 (3)	0.210
CCS	154 (61)	70 (54)	84 (69)	0.021
Complexity of disease, *n* (%)				
1VD	140 (56)	80 (62)	60 (49)	0.065
2VD	66 (26)	32 (25)	34 (28)	0.657
3VD	35 (14)	12 (9)	23 (19)	0.043
LMT	11 (4)	6 (5)	5 (4)	1.000
CTO, *n* (%)	37 (15)	13 (10)	24 (20)	0.047
DES-hybrid treatment, *n* (%)	60 (24)	26 (20)	34 (28)	0.188
Echocardiographic data				
LVEF, %	54.1 ± 13.5	57.7 ± 11.7	50.2 ± 14.3	<0.001
Biochemical data				
Sodium, mmol/L	140.1 ± 3.0	140.2 ± 2.6	140.0 ± 3.3	0.536
Potassium, mmol/L	4.3 ± 0.5	4.2 ± 0.4	4.3 ± 0.5	0.022
Chloride, mmol/L	104.6 ± 3.1	104.5 ± 2.7	104.6 ± 3.5	0.864
Medications, *n* (%)				
Aspirin, *n* (%)	230 (91)	118 (91)	112 (92)	0.946
P2Y_12_ Inhibitors, *n* (%)	235 (93)	119 (92)	116 (95)	0.385
Oral anticoagulant agents, *n* (%)	43 (17)	21 (16)	22 (18)	0.819
Statins, *n* (%)	215 (85)	115 (89)	100 (89)	0.201
RASi, *n* (%)	182 (72)	99 (76)	83 (74)	0.194
MRA, *n* (%)	34 (13)	12 (9)	22 (18)	0.040
β blockers, *n* (%)	150 (60)	66 (51)	84 (69)	0.005
SGLT2 Inhibitors, *n* (%)	44 (17)	23 (18)	21 (17)	0.920

AF, atrial fibrillation; CAD, coronary artery disease; MI, myocardial infarction; PCI, percutaneous coronary intervention; CABG, coronary artery bypass graft; CKD, chronic kidney disease; PAD, peripheral artery disease; AAA, abdominal aortic aneurysm; COPD; chronic obstructive pulmonary disease; STEMI, ST-segment elevation myocardial infarction; NSTEMI, non-STEMI; CCS, chronic coronary syndrome; VD, vessel disease; LMT, left main coronary trunk; CTO, chronic total occlusion; DES, drug eluting stent; LVEF, left ventricular ejection fraction; RASi, renin angiotensin system inhibitor; MRA, mineralocorticoid receptor antagonist; SGLT2, sodium glucose cotransporter 2.

**Table 2:** Comparison of clinical outcomes according to CKD status.

	No CKD (*n* = 130)	CKD (*n* = 122)	HR (95% CI)	*p* Value	Adjusted HR (95% CI)	*p* Value
All-Cause Death	3 (2.3%)	16 (13.1%)	5.689 (1.656–19.540)	0.006	4.880 (1.337–17.804)	0.016
Cardiovascular Death	0 (0%)	3 (2.5%)	NA	NA	NA	NA
Any Myocardial Infarction	3 (2.3%)	9 (7.4%)	3.282 (0.889–12.140)	0.075	3.217 (0.770–13.450)	0.109
Target Vessel Revascularization	17 (13.1%)	17 (13.9%)	1.081 (0.552–2.119)	0.820	1.207 (0.576–2.531)	0.618
Stroke	1 (0.8%)	3 (2.5%)	3.390 (0.352–32.620)	0.290	1.226 (0.102–14.694)	0.872
Bleeding	0 (0%)	1 (0.8%)	NA	NA	NA	NA

CKD, chronic kidney disease; HR, hazard ratio; CI, confidence interval; NA, not applicable.

## Data Availability

The datasets generated and analyzed in the present study are not publicly available but are available from the corresponding author upon reasonable request.

## Supplementary Materials

The following supporting information can be downloaded at: https://www.mdpi.com/article/10.3390/jcm14072317/s1, Figure S1: Cumulative incidence of composite events according to CKD status in patients with (A) ACS, and (B) CCS. CKD, chronic kidney disease; ACS, acute coronary syndrome; CCS, chronic coronary syndrome. Figure S2: Cumulative incidence of composite events according to CKD stage. CKD, chronic kidney disease.
