# Supplementary material for: Prognostic Impact of Chronic Kidney Disease After Percutaneous Coronary Intervention with Drug-Coated Balloons
Source: J Clin Med. 2025 Mar 28;14(7):2317. doi: 10.3390/jcm14072317 (PMC11989811; doi:10.3390/jcm14072317)
Supplement: Supplementary file 1 [file jcm-14-02317-s001.zip › jcm-3516328-supplementary.pdf]

Supplemental Figure S1. Cumulative incidence of composite events according to CKD status in patients with (A) ACS, and (B) CCS. CKD, chronic kidney disease; ACS, acute coronary syndrome; CCS, chronic coronary syndrome.

Supplemental Figure 1.

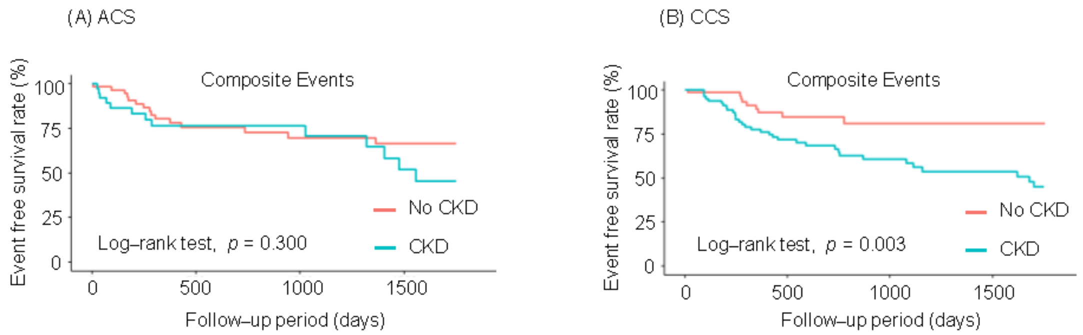

No at risk

No CKD	60	29	23	19
CKD	38	17	13	8

No at risk

No CKD	70	32	20	16
CKD	84	46	28	21

Supplemental Figure S2. Cumulative incidence of composite events according to CKD stage.

CKD, chronic kidney disease.

Supplemental Figure 2.

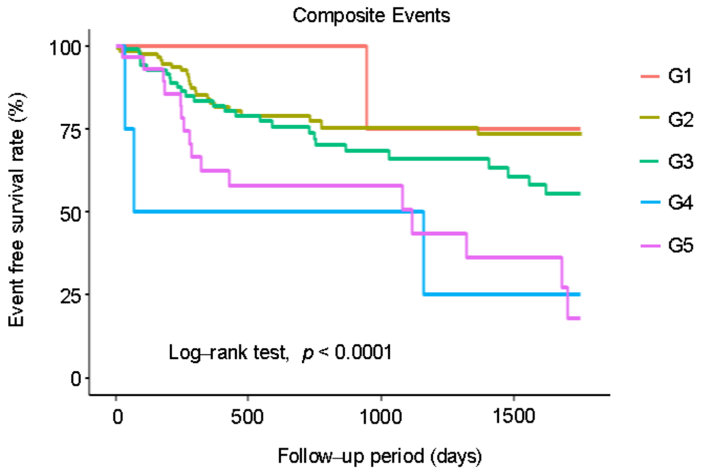

No at risk

G1	11	6	3	3
G2	119	55	40	32
G3	88	49	31	23
G4	4	2	2	1
G5	30	12	8	5
